# On the Relation between the Small World Structure and Scientific Activities

**DOI:** 10.1371/journal.pone.0121129

**Published:** 2015-03-17

**Authors:** Ashkan Ebadi, Andrea Schiffauerova

**Affiliations:** 1 Concordia Institute for Information Systems Engineering (CIISE), Concordia University, Montreal, Quebec, Canada; 2 Department of Engineering Systems and Management, Masdar Institute, Masdar City, Abu Dhabi, United Arab Emirates; Max Planck Society, GERMANY

## Abstract

The modern science has become more complex and interdisciplinary in its nature which might encourage researchers to be more collaborative and get engaged in larger collaboration networks. Various aspects of collaboration networks have been examined so far to detect the most determinant factors in knowledge creation and scientific production. One of the network structures that recently attracted much theoretical attention is called small world. It has been suggested that small world can improve the information transmission among the network actors. In this paper, using the data on 12 periods of journal publications of Canadian researchers in natural sciences and engineering, the co-authorship networks of the researchers are created. Through measuring small world indicators, the small worldiness of the mentioned network and its relation with researchers’ productivity, quality of their publications, and scientific team size are assessed. Our results show that the examined co-authorship network strictly exhibits the small world properties. In addition, it is suggested that in a small world network researchers expand their team size through getting connected to other experts of the field. This team size expansion may result in higher productivity of the whole team as a result of getting access to new resources, benefitting from the internal referring, and exchanging ideas among the team members. Moreover, although small world network is positively correlated with the quality of the articles in terms of both citation count and journal impact factor, it is negatively related with the average productivity of researchers in terms of the number of their publications.

## Introduction

The world is really small! This comes to our minds when a mutual acquaintance is found with someone who we do not know at all. The idea of the small world network is traced back to the work of Milgram in 1967. Through a series of field experiments he found that even in a very large network on average only six intermediates are needed to reach a person who is completely unknown. This property is also called “six degrees of separation” in the literature [1]. Later, Travers and Milgram [2] tried to formulate the small world property by calculating the probability of any two randomly chosen people knowing each other in a large population. In other words, in small world networks the average path length (average distance between two given nodes in the network) is relatively short in spite of the existence of high clustering (tendency of the nodes in a network to cluster together). Therefore, short path lengths among network actors facilitate the spread of various ideas that are generated in separate clusters, which results in producing novel knowledge [3,4].

The level and the efficiency of knowledge diffusion are affected by small world property. Cowan and Jonard [5] developed a model to study the efficiency of the small world networks and claimed that the level of knowledge is at its maximum when the network structure has small world properties. Therefore, it is good to have small world property in the network but how persistent are such networks? Kogut and Walker [6] analyzed the cross-ownership among German firms during 1990s and the robustness of the small world property. They found that the small world network tends to preserve its properties of high clustering and short path lengths even if it experiences a considerable number of shocks and re-structuring of the links of the network. Therefore, once the small world network is established it retains the property unless the network perceives a considerable amount of re-structuring forcing it to transform into another structure.

Several researchers analyzed the effect of small world property in the network of firms. Sullivan and Tang [7] constructed the inter-firm network of the United States venture capital industry to evaluate its effects on the firms’ performance. They observed a positive impact of the small world structure on productivity of the firms. In another study, Baum *et al*. [8] investigated the Canadian network of investment bank syndicate from 1952 to 1990 to see how small world network emerges and evolves over time. They confirmed that the networks formed among firms usually resemble small world characteristics. Schilling and Phelps [9] focused on the impact of small world property on firms’ performance through analyzing the number of patents. Their results show that there is a positive effect of the small world since high clustering and short path length enable companies to get access to new knowledge that is required for innovation.

In addition, several empirical studies focused on individuals’ activity and analyzed the effect of the small world property on the performance of individuals in the network. Fleming and Marx [4] studied the collaboration of the inventors in Silicon Valley and Route 128 in Boston and found that the network of the examined inventors resembled the small world structure. However, no positive relation was observed between the existing small world property in the network and the inventive productivity of the researchers in the region. Fleming *et al*. [10] have also shed some light on the impact of the small world in the network of inventors and their innovative and managerial approaches within a small world network to remain competitive. Although they found a positive effect of short average path length on the technological productivity, no significant positive influence of the small world property was observed.

Other studies analyzed the impact of the small world structure in co-authorship networks. Co-authorship analysis has been particularly recognized by some studies (*e*.*g*. [11,12]) as being the most common tool in investigating the relations and patterns in scientific collaboration. Newman [13] investigated the co-authorship networks in physics, biology and mathematics and found the small world structure in all the aforementioned networks. Goyal *et al*. [14] focused on a single scientific discipline. Using the co-authorship network of economists during 1980 to 1999, they found small world properties in the examined collaboration network. Moreover, they found an increasing trend in the average degree of the network over time and realized that the number of brokers is also augmenting. In another study, despite considering several fields for the study Moody [15] also focused on the subspecialties (*e*.*g*. economic sociology, criminology, *etc*.) in a single discipline and analyzed the network of sociologists during the period of 1963 to 1999. He surprisingly found that the network did not resemble the small world properties likely due to the considerable overlap among the subfields and the authors.

Hence, there is a tendency in co-authorship networks for the small world structure. Role of the best connected actors in joining the other individuals and clusters in the network is very important. Moreover, the co-authorship pattern in a scientific field is also crucial for a network to obtain small world structure. The more a scientific discipline is team oriented and the larger the size of the team, the more probability of finding the small world properties in the structure [16,17]. Therefore, the analysis of small world properties is more seen in the disciplines in which teamwork is common [18].

Studies that have generally assessed impact of the network structure variables in co-authorship networks have found correlations between the centrality measures and some performance variables [19–22]. Yan and Ding [19] focused on 16 journals in the field of library and information science (LIS) and constructed the co-authorship network at the micro level over the time span of 1988 to 2007. They calculated four centrality measures for the authors in the network, *i*.*e*. betweenness centrality, degree centrality, closeness centrality and PageRank and found a positive relation between the mentioned measures and citation counts of articles. Abbasi *et al*. [20] focused on the scholars in the field of information systems and statistically analyzed the impact of the network structure variables on the performance of the researchers using citation based indicators. They found a positive relation between all the network structure variables and the performance of the scholars except for the betweenness and closeness centralities. In another study, Kumar and Jan [23] assessed and compared the impact of the network variables in the field of energy fuels on research performance in Turkey and Malaysia. According to their results, popularity, position and prestige of the researchers measured by the network centrality indicators have a positive impact on their research performance. In addition, they found PageRank as the most influential centrality measure. Eslami *et al*. [22] focused on the field of biotechnology in Canada and statistically investigated the impact of the network structural variables on the quantity and quality of technological performance of the researchers within the period of 1966 to 2005. Their results suggest a significant impact of the structure of the examined co-authorship network on knowledge and technology production, however, no impact was observed on the quality of the patents.

Nevertheless, the results about the impact of the small world structure on performance are inconsistent. For example, Fowler [24] found a non-linear relation between small world properties and voting participation rate, and Uzzi and Spiro [3] found a similar relation between the financial and artistic performance of the artists and the small world properties. However, Schilling and Phelps [9] observed a linear relation whereas Fleming *et al*. [10] found no relation between small world properties and performance. Hence, no consensus is found in the literature about the impact of the small world structure on the performance [25]. One reason could be the use of different datasets and performance measures in the studies that makes it hard to come into a general agreement about the impact of the small worldiness on researchers’ performance. Hence, the assessment of the impact is suggested to be done in different fields and scientific environments. In addition, although there are very few studies that particularly analyzed the impact of the small world variables on productivity of the inventors and firms, to our knowledge no study has analyzed its relation with the quality of publications and researchers’ team size. This paper is designed to fill these research gaps.

Our main objective is first to analyze if the examined network resembles the small world property and then to study its relation with the scientific output, the quality of the produced papers and the team size. It is assumed that analyzing the relation between the small world property and the quality of the publications will help to highlight the benefits of a systematic collaboration network rather than a random one in producing higher quality research. In addition, it will identify the importance of a well-established collaboration network in which researchers are well connected by short distances. Moreover, analyzing the relation between the small world property and the average team size of the researchers will determine if researchers in a small world network have larger team sizes due to the shorter distance among researchers in such a network. As being a member of a larger team size may result in higher rate of publication for each of the team members, if a positive relation is observed between the small world property and the team size then one may expect higher overall rate of publications in such collaboration networks.

In order to achieve this objective a comprehensive dataset of publications of Canadian researchers in natural sciences and engineering was used. First the existence of the small world properties in the co-authorship network of these researchers was examined and then the inter-relations between the small world variables and quantity of the scientific output (measured by the number of publications), quality of the articles (measured by the normalized citation rate and by the average impact factor of the journals) and size of the research teams (represented by the average number of authors per paper) were statistically investigated. The rest of the paper is organized as follows: Section “Data and Methodology” describes methodology and data used in this study. The empirical results and interpretations are provided in section “Results”. Section “Conclusion” presents the findings of this research and the limitations of this study are discussed in the last section “Limitations”.

## Data and Methodology

The study has three phases. In the first phase, a database of all the research publications produced by the Canadian researchers in natural sciences and engineering was created. It was decided to focus only on engineering and natural sciences and to exclude social and medical sciences, because collaboration patterns in different disciplines vary (as an example, please see [26]). In order to do so only the researchers funded by Natural Sciences and Engineering Research Council (NSERC), which is the main Canadian federal funding agency for the researchers working in all the areas of engineering and natural sciences, were included. Since almost all the Canadian researchers in these research fields are currently receiving or received in the past a research grant from NSERC [27], it was assumed that this approach allows us to identify them quite effectively. This procedure was more straightforward than collecting all the Canadian papers and trying to distinguish between the ones that are written by the researchers in natural sciences and engineering and other scientific fields through employing some keywords or journal categories. Eligibility for NSERC funding makes our target researchers clearly defined. The funding data was collected from NSERC website. Then the articles written by these researchers were collected from SCOPUS (*i*.*e*. a commercial database of scientific articles that has been launched by Elsevier in 2004) within the period of 1996 to 2010 since the data quality of SCOPUS was low before 1996 (*e*.*g*. lack of the citation data before 1996). Moreover, to have a proxy of the quality of the papers SCImago was used to collect the impact factor information of the journals in which the articles were published. SCImago was chosen for two main reasons. Firstly, it provides annual data of the journal impact factors that enabled us to perform a more accurate analysis since the impact factor of the journals are considered in the year that an article was published not its impact in the current year. Secondly, SCImago is powered by SCOPUS that makes it more compatible with our articles database. In total, the final database contained 130,510 articles and 177,449 authors together with all the related information (*e*.*g*. article title, co-authors, their affiliations, year of publication).

In the second phase, Pajek software was used to construct the collaboration networks of the researchers and to measure the structural network and small world variables. Co-authoring an article was assumed as a sign of collaboration among the researchers, but there was no information on the length of this relationship. In some of the similar studies (*e*.*g*. [10,28]) a 5-year period for the life of each created collaboration link in the networks was considered while in other studies a 3-year time window has been assumed (*e*.*g*. [29]). The indicators were calculated for both of the mentioned time windows and it was found that the results are more robust for the 3-year time window. In other words, several independent variables were considered at first as the candidates for the control variable. Then correlations among various combinations of dependent and independent variables were tested to select the model with no significant correlation among the variables. Moreover, 3-year and 5-year time windows were considered for constructing the co-authorship networks. Since the number of observations dropped for the 5-year time window, we could not fit a model with no significant correlation among the variables. Therefore, we selected the 3-year time window. Hence, a 3-year time window was assumed in our study and the 3-year moving window was shifted forward from 1996 to 2010 to extract the publications for each of the networks. This procedure resulted in 12 undirected networks. The structure of the 12 networks was then analyzed separately by Pajek software to measure the small world variables for each of the 12 networks.

In the last phase, the measures calculated in the previous phase were used as inputs to statistically analyze the inter-relations between small world properties, the productivity and scientific collaboration of the scientists. For this purpose, five regression models were defined and estimated by STATA. The first dependent variable accounts for the research productivity of the researchers within each of the 12 periods (*no_art*). The number of publications has been widely used in the literature as a proxy of the scientific productivity (*e*.*g*. [30,31]). A single year for representing the productivity of the researchers was considered since it is assumed that the results of researchers’ collaboration come to light soon after the respective collaboration period is finished (as was done in [10,28]). In other words, it is assumed that the 3-year collaborative activity among the researchers will be reflected in the next year in the form of the number of their publications. Hence, for the total number of articles in the year *i* (*no_art*_*i*_), the small world variables were calculated for the networks constructed on the 3-year snapshot from year *i-3* to *i-1*. To further investigate, the number of publications was normalized by dividing it by the number of authors and was considered as the dependent variable for the second regression model (*art_per_aut*_*i*_). This helped us to better analyze the relation between the small world variables and productivity since higher number of authors may result in higher number of publications. Hence, by averaging the number of publications over the number of co-authors the impact of the raise in the number of authors was accounted. In order to assess the quality of the publications the normalized number of citations was used in the third model. Citation count based indicators are one of the most widely used approaches in determining research quality [32]. However, like all the methods they have some drawbacks, *e*.*g*. negative citations, self citations [31], and limitations of the citation data source [33]. Nevertheless, it is generally accepted in bibliometrics that the real or expected number of citations received by publications can be used as a good index of the mean impact at the aggregate level [34,35]. Hence, the citation counts were normalized based on the following definition and were used for the analysis at the aggregate level:

$${\mathrm{ncit}}_{i}=\frac{\mathrm{total citation count in year i}}{\left(2010-\mathrm{year i}+1\right)\;*\;\mathrm{number of papers in year i}}$$

where (2010—year *i* + 1) represents the gap between the current year and the final year of the study and is used for normalizing the citation counts. The reason for normalizing the number of citations is that older articles have more chance to be cited. Hence, in general as we move toward the recent periods the total number of citations decreases. The average impact factor of the journals in which the articles were published was also used as another proxy for the quality of the papers and defined the fourth dependent variable (*avgif*). The last dependent variable represents number of authors per article in year *i* (*aut_per_art*_*i*_) as a measure for the team size of the researchers.

The independent variables that were considered in all the five aforementioned models are as follows:

- Small World (*sw*)
- Network Connectivity (*netcon*)

In order to calculate the small world variable, it was needed to calculate clustering coefficient and average path length. In the following, the definitions of the clustering coefficient and path length along with the independent variables’ definitions are presented.

### Clustering Coefficient (CC)

This index counts the number of triangles in the given undirected graph to measure the level of clustering in the network. In other words, it is the likelihood that two neighbors of a node in a graph are connected to each other; hence it measures the tendency of the nodes to cluster together [36]. According to Watts and Strogatz [37] the clustering coefficient can be defined based on a Local Clustering Coefficient (LCC) for each node within a network. LCC is defined as follows:

$${\mathrm{LCC}}_{i}=\frac{\mathrm{number of triangles connected to node i}}{\mathrm{number of triples centered on node i}}$$

The denominator of the above formula counts the number of sets of two edges that are connected to the node *i*. The overall clustering coefficient is calculated by taking average of the local clustering coefficient of all the nodes within the network. Hence,

CC=1n∑i=1nLCCi

in which *n* denotes the number of vertices in the network. This measure returns a value between 0 and 1 in a way that it gets closer to 1 as the network interconnectivity increases.

### Shortest Path Length (PL)

**Fig 1:**
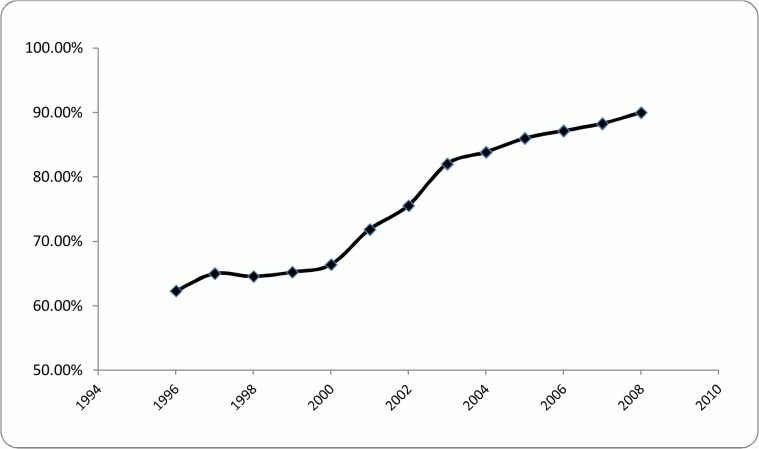
Historical trend of largest component proportion.

This index represents the separation degree of the network and is the lowest number of vertices that are needed to be traversed to reach from one vertex to another vertex [38]. The shorter the distance is the more easily information may flow among the researchers. The path length was calculated for the largest component of each of 12 created co-authorship networks. Component of a network is a sub-network in which there is no isolated vertex and all the vertices are interconnected. From the definition, the small world variable is measured for the largest component of each network. This limitation is due to the fact that the shortest path can be calculated just in a connected network. Hence, the largest connected component was considered for measuring the aforesaid variable in each of the 12 generated networks. This assumption has been widely employed in the literature (*e*.*g*. [3,10,28,39–41]) and is justifiable, since the core research activities mainly occur in the largest component in which the most influential authors are present [42]. Moreover, the proportions of the largest component in our created networks are not only large in comparison with similar studies (*e*.*g*. [21,41,43,44]), but they are even gradually increasing. After 2002 our largest component covered more than 75% of the whole network, reaching to the level of almost 90% in the last period (Fig. 1). Therefore, the largest component can be used for the calculation of the path length.

### Small World (SW)

The small world variable is calculated based on the clustering coefficient and the path length:

SW=CCPL

### Network Connectivity (netcon)

It is a measure of the connections between pairs of vertices and is related to the average degree of the network. In other words, in the co-authorship network of the researchers it indicates the average number of collaborators for each researcher who had at least one article co-authorship during the given period of time. This is an important measure since higher number of co-authors in a network results in a tighter network that facilitates the knowledge exchange [45]. The network connectivity (*netcon*) was used as the control variable. The reason is that higher number of researchers in a network can increase the chance of higher network connectivity and consequently the chance of higher collaboration among the researchers that may have an effect on our dependent variables.

## Results

### Pre-analysis

**Fig 2:**
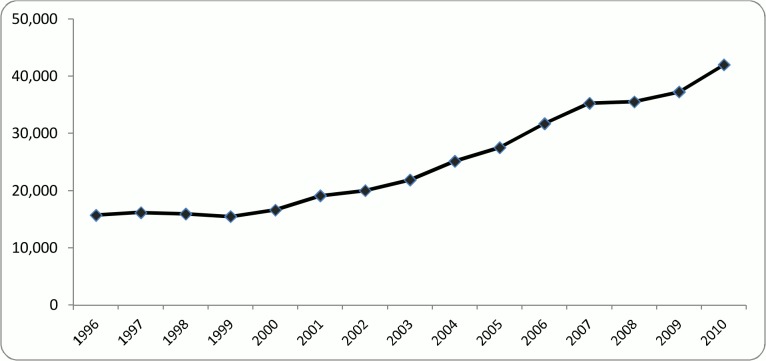
Historical trend of the researchers from 1996 to 2010.

Number of the researchers in each of the examined periods of time reflects the size of the network in the corresponding year. As the first step, the trend of the network size was analyzed. According to Fig. 2, the network size did not change much until 2000 since then it has been steadily increasing with an almost constant positive slope. Since an annual increase was expected in the number of researchers, the steady line indicating the number of researchers between 1996 and 2000 might be due to the SCOPUS data that seems to be more integrated and complete for the recent years. Another reason for the steady trend during the first five years could be the immaturity of the examined collaboration network in a way that after a couple of years new researchers started joining the network with a faster pace. This issue will be further investigated in the rest of the paper.

**Fig 3:**
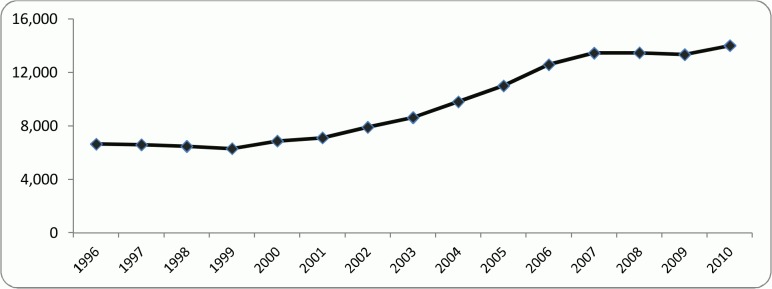
Historical trend of the researchers’ articles from 1996 to 2010.

In line with the increase in the number of authors an increase is seen in the number of articles, having almost the same trend. According to Fig. 3, the number of articles remained constant during the first and the last 5-year periods. However, a positive jump is observed during the second 5-year period (from 2001 to 2005).

### Small world analysis

**Fig 4:**
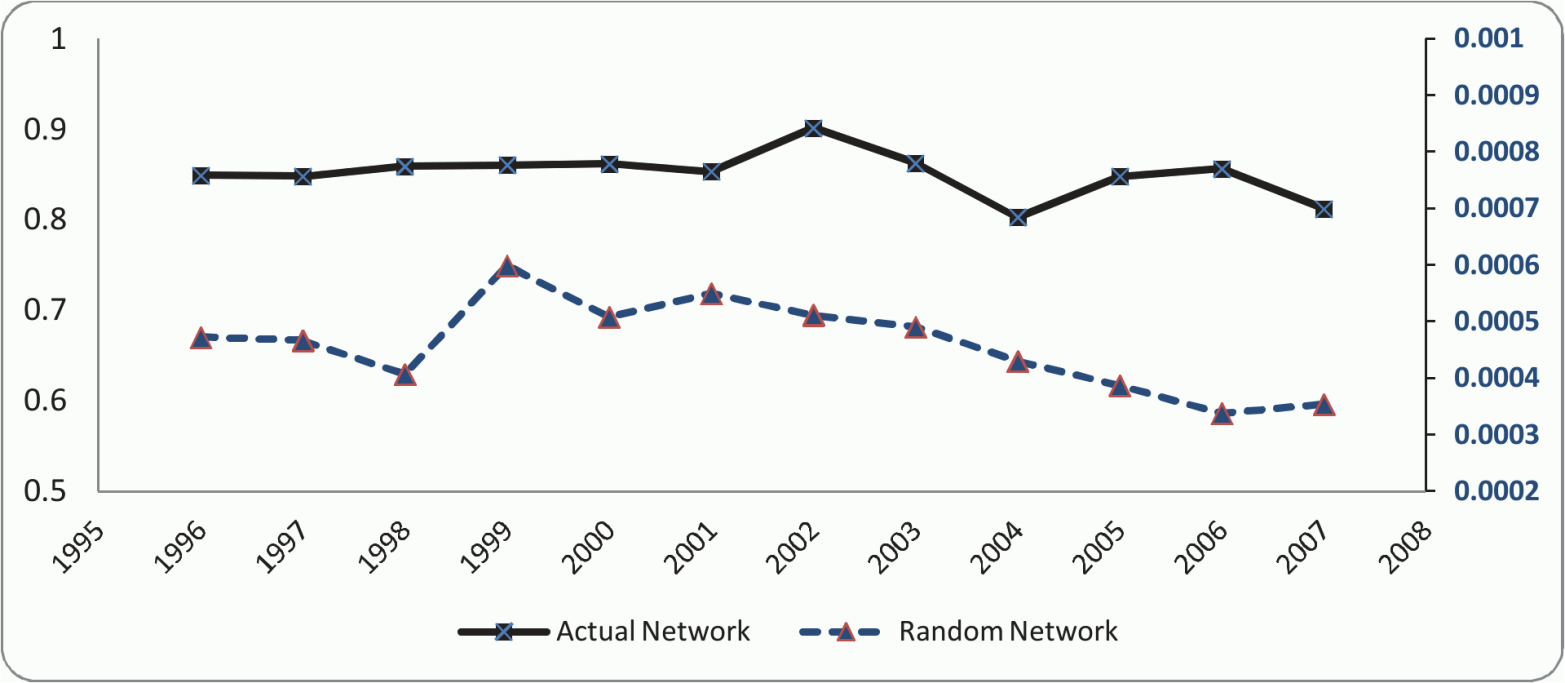
Clustering coefficient, actual and random networks.

**Fig 5:**
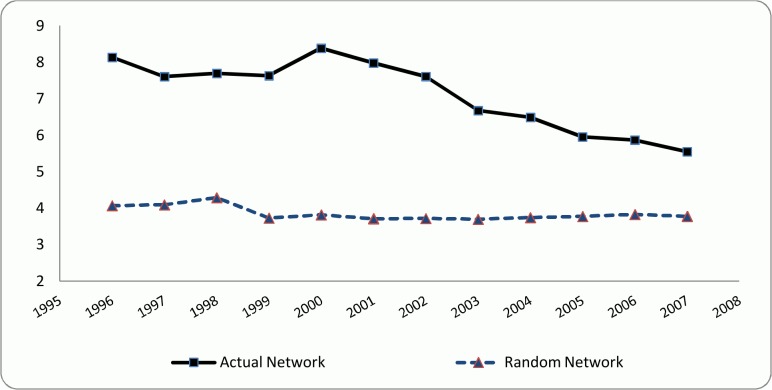
Path length, actual and random networks.

According to Kogut and Walker [6], a network has a small world structure if its average clustering coefficient is significantly higher than a random network of the same number of vertices while having approximately the same path length. Hence, in order to investigate the small world structure in the co-authorship network of the researchers, an Erdős–Rényi random network [46] of the same size as the actual network was constructed for each of the examined periods. The respective path lengths and clustering coefficients were then calculated for the generated random networks and compared to the corresponding amounts of the actual networks. The results are depicted in Fig. 4 and Fig. 5. The X-axis in Figs. 4 and 5 represents the starting year of each of the 3-year time intervals that were considered to calculate the collaboration network variables. For example, 1996 represents the period of [1996–1998].

Although the small world networks are often large in size, they exhibit relatively short path length and high clustering coefficient [47]. Clustering coefficient in co-authorship network represents if researchers’ collaborators are also collaborating with each other in form of writing a paper jointly [48]. As it can be seen in Fig. 4, the clustering coefficient for the actual network is almost constant maintaining about 0.8 and is significantly higher than the clustering coefficient for the respective random networks (that are between 0.0003 and 0.0006) in all the examined periods. This result is completely in line with the previously done studies that investigated the small world structure (*e*.*g*. [43,48]). This is a primary sign of the small world structure in the examined network of researchers. In addition, the clustering coefficient of the examined network is very high in comparison with the other similar studies, *e*.*g*. all the four co-authorship networks studied by Newman [49], and SIGMOD co-authorship networks of Nascimento *et al*. [44]. This indicates that in the examined network it is more likely for two co-authors to have a common collaborator with whom they have also published an article.

The path length for the actual and the generated random networks were compared. According to Fig. 5, although the path length of the examined co-authorship network remains relatively constant during the initial 5-year period, it starts dropping significantly and continuously after 2000, while getting very close to the path length of the random network. The value of the path length of our examined network is almost similar to the one of Nascimento *et al*. [44] who found a path length of 5.65 in the SIGMOD co-authorship network, and is lower than some other studies (*e*.*g*. [41]). In general, in other similar studies that contain more than 10,000 vertices and analyzed the small world property in co-authorship networks, the average path length is not more than 10 (*e*.*g*. [50,51]). According to Fig. 4 and Fig. 5 and based on the definition of Watts and Strogatz [37] the examined co-authorship network of researchers strictly resembles the small world structure.

As the next step, SW indicator was defined and used to analyze the small world characteristics of the collaboration network of researchers. To calculate the value of the small world indicator the method that has been employed in several similar studies was followed (*e*.*g*. [6,28,52]) which used the following formula for calculating the small world ratio:

SW=CCaCCrPLaPLr

Table 1 shows the results for the small world variables calculated for all the examined periods. According to Baum *et al*. [28], as the size of the network increases the value of the small world indicator should increase. As it can be seen in Table 1, there is an increase in the amount of SW indicator during the first three periods. After a sudden drop, it continues to increase steadily after 1999 reaching to the maximum value of the SW indicator in the latest periods. The drop could be due to two reasons. First, the SCOPUS data was probably less complete during the first intervals as the number of articles found in SCOPUS is almost constant in the first three periods. Second reason could be the nature of the collaboration network that may have been less mature during the initial periods. As more researchers join the network, more links are established and the network evolves dynamically. This enables the network to reflect more small world properties as the time passes. This proposition is also confirmed by the trend of the clustering coefficient.

**Table 1:** Small world characteristics for the collaboration network.

		Actual to Random Ratio		
Period	Network Size	Path Length	Clustering Coefficient	SW
**[1996–1998]**	32,862	2.00	1798.74	899.12
**[1997–1999]**	33,111	1.86	1817.13	977.91
**[1998–2000]**	33,931	1.80	2113.11	1,175.71
**[1999–2001]**	36,700	2.05	1436.80	701.86
**[2000–2002]**	39,870	2.20	1697.40	772.46
**[2001–2003]**	43,348	2.15	1553.13	722.69
**[2002–2004]**	47,793	2.05	1762.30	860.31
**[2003–2005]**	53,191	1.81	1760.39	974.64
**[2004–2006]**	59,427	1.73	1868.85	1,077.50
**[2005–2007]**	65,344	1.58	2192.22	1,388.19
**[2006–2008]**	69,868	1.53	2538.09	1,655.32
**[2007–2009]**	73,518	1.47	2295.90	1,562.00

**Fig 6:**
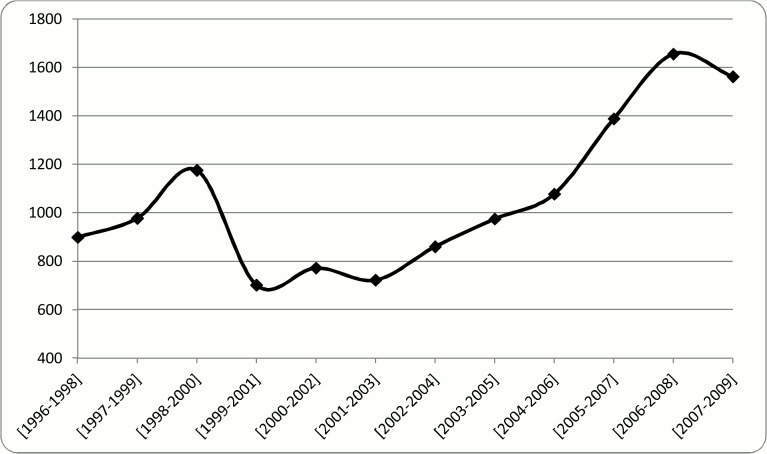
Small world trend.

However, it is also argued in the literature that small world properties follow the form of an inverted U-shape (*e*.*g*. [53]). That means an increase in the small world properties will be followed by a later decrease. According to Fig. 6, the trend of SW indicator in the examined network had a local maximum in the period of [1998–2000] and then after a sudden decline it started to rise again till the period of [2006–2008] where the second local maximum is seen. Hence, a declining trend is expected to be seen after 2007 and a reassessment of the small world properties is suggested for the future. In a small world network, researchers can get access to the pools of knowledge in diverse clusters and communities through knowledge brokers who are the actors in the network that connect different clusters. Therefore, other actors can retain or even improve their position in the network by accessing continuously to the flows of diverse information and knowledge or other resources [54,55]. The reason for the inverted U-shaped form of the small world property is that as the network evolves the knowledge brokers become less important gradually due to the limited advantages of the brokerage positions that will lead to the decline of the small world. In other words, as the network evolves different clusters gradually get familiar with the information pools of the other clusters through the existing knowledge brokers, hence making the knowledge generated in different clusters more homogeneous. Facilitating the knowledge exchange reduces the diversity in the whole network gradually [56], making the role of the knowledge brokers less important. As a result of the decline in the entrance of new knowledge brokers along with the decay of the old brokers, network becomes more separated. Hence, actors collaborate with their stable and familiar partners within their own clusters and communities. This will lead to multiple isolated clusters and consequently lower small-worldiness [53].

To compare the small world structure in the examined collaboration network a list of previously identified small world co-authorship networks as well as the network properties is presented in Table 2. Considering the network size, the NSERC researchers’ co-authorship network is similar to the SPIRES and LANL co-authorship networks of Newman [49], and MATH co-authorship network of Barabási *et al*. [48]. NSERC network is significantly more cliquish (*i*.*e*. it has a very high respective clustering coefficient) than SPIRES network where the value is quite comparable to the one for the LANL network. However, it is less cliquish than the MATH network that has the highest clustering coefficient among all the listed networks. Comparing the path length of our examined network with the mentioned networks, it can be said that NSERC network is more similar to the LANL network of Newman [49]. As it can be seen in Table 2, all the previously studied small world networks have the path length ratio lower than 2, ideally closer to 1. In the case of our examined co-authorship network the path length ratio is declining and getting very close to 1 in the final period (1.47). However, different clustering coefficient ratios are observed in the previous studies that led them to a wide range of values for the SW indicator.

**Table 2:** Comparison of previously studied co-authorship networks with the last period of our network (NSERC).

		Actual to Random Ratio			
Network	Network Size	Path Length	Clustering Coefficient	SW	Reference
**SIGMOD co-authorship**	1,413	1.33	172.5	129.7	Nascimento *et al*. [44]
**NCSTRL co-authorship**	11,994	1.16	1653.34	1425.3	Newman [49]
**LANL co-authorship**	52,909	1.23	2388.9	1942.2	Newman [49]
**SPIRES co-authorship**	56,627	1.89	242	128.05	Newman [49]
**Math co-authorship**	70,975	1.16	10925.93	9418.91	Barabási *et al*. [48]
**Sociologists co-authorship**	128,151	1.30	0.94	0.72	Moody [15]
**MEDLINE co-authorship**	1,520,251	0.94	6000	6382.98	Newman [49]
**NSERC co-authorship**	73,518	1.47	2295.90	1,562.0	

### Statistical Analysis

After observing the small world structure in the examined co-authorship network, the inter-relations between the small world property and several bibliometric measures were statistically analyzed. As the first step, any pair wise correlations among the independent variables were checked and no significant correlation was found among them.

Negative binomial regression model was considered for our first dependent variable, *i*.*e*. number of articles in the following year. Since the dependent variable in the first model is a count measure, the best regression model would be the Poisson model [57]. However, for a Poisson regression the variance and mean of the sample should not differ significantly. Hence, the data should be tested to detect any over-dispersion or under-dispersion that will lead the Poisson model to underestimate or overestimate the standard errors resulting in misleading estimates for the statistical significance of variables [58]. Therefore, the likelihood ratio test was done to see if the Poisson model fits our data. The results show that the over-dispersion coefficient (α) is significantly different from zero, which means that Poisson distribution is not an appropriate choice and negative binomial regression could be a better estimator. For the remaining 4 dependent variables, *i*.*e*. normalized citation count in the following year, average impact factor of journals in which the articles have been published in the following year, number of articles per author in the following year, and number of authors per article in the subsequent year, linear regression models were used.

Table 3 shows the results for the inter-relation analysis between the small world property and productivity of the researchers in terms of the number of their publications. The results show that both of the independent variables (small world and network connectivity) can be regarded as significant predictors of the scientific productivity in the following year.

**Table 3:** Regression results for number of articles model.

Negative binomial regression				Number of obs		= 12
				LR chi2(2)		= 38.39
Dispersion		= mean		Prob > chi2		= 0.0000
Log likelihood		= −93.176699		Pseudo R2		= 0.1708
**no_art**	**Coef.**	**Std. Err.**	**z**	**P > |z|**	**[95% Conf. Interval]**	
*SW*	.0003522	.0000558	6.32	0.000	.0002429	.0004615
*netcon*	.0269058	.0019361	13.90	0.000	.0231111	.0307005
_*cons*	7.658743	.0934756	81.93	0.000	7.475534	7.841951
/*lnalpha*	−5.757512	.4220285			−6.584673	−4.930351
*alpha*	.003159	.0013332			.0013814	.007224
Likelihood-ratio test of alpha = 0: chibar2(01) = 327.04				Prob> = chibar2 = 0.000		

According to the results, the small world property and network connectivity are positively correlated with the number of publications of the researchers in the subsequent year. These results were expected since as the network becomes more connected, researchers get more familiar with other scientists’ fields of research that may lead to the establishment of more collaboration links. In addition, the small world structure can accelerate the exchange of knowledge and expertise among the researchers that may result in higher productivity. The reason is that small world networks allow access to distant information and the knowledge is transferred more efficiently in such networks [25]. Our results are in accordance with major conclusions of the previous studies (*e*.*g*. [5,6,22]).

Collaboration among the researchers in a small world network was also tested. For this purpose, the number of authors per articles was considered as a proxy of the researchers’ team size and its relation with the small world structure was assessed. Number of authors per article is a common indicator in scientometrics and has been widely used in the literature as a proxy for scientific collaboration (*e*.*g*. [59,60]). As it can be seen in Table 4, only the small world variable is significant reflecting a small positive relation with the team size. Hence, it seems that researchers might benefit from the shorter path length and more clustered sub-networks to get in touch with other researchers who are working in the same scientific area. This may result in establishment of new collaboration links and expansion of their team size. Moreover, high clustering creates more repeated links among the researchers, causing the risk to be shared among the researchers that might lead to an increase of the trust level in the community [61]. Of course other factors like changes in society, funding, regulations, *etc*. can also play a role here. In addition, it may also possible that the author effect is a cause and the small world effect is a result. As the next step, relation with the average productivity of the researchers was assessed.

**Table 4:** Linear regression results for team size model.

Source	SS	df	MS		Number of obs	= 12
Model	.194938377	2	.097469189		F (2, 9)	= 9.74
Residual	.090073661	9	.010008185		Prob > F	= 0.0056
Total	.285012038	11	.025910185		R-squared	= 0.6840
					Adj R-squared	= 0.6137
					Root MSE	= .10004
**aut_per_art**	**Coef.**	**Std. Err.**	**t**	**P > |t|**	**[95% Conf. Interval]**	
*SW*	.0003812	.0000967	3.94	0.003	.0001624	.0006
*netcon*	.0034309	.0034135	1.01	0.341	−.004291	.0111527
_*cons*	2.050345	.1626485	12.61	0.000	1.682408	2.418281

Since the number of authors has an increasing trend over the examined period, to assess the productivity of the researchers more accurately the average number of articles per author was examined. The result of the linear regression model is depicted in Table 5. As it can be seen the small world property is negatively correlated with the average productivity of the researchers, which is an interesting finding. Although a positive relation was found between the small world structure and the total number of articles, it is observed that it may harm the average publication rate. Hence, it seems that in a small world structure researchers start to collaborate more by forming bigger scientific teams that may lead them to increased overall productivity. However, when it comes to the average productivity per researcher it becomes lower since the team sizes have grown. The other aspect to be analyzed is the quality of the papers that are produced. Therefore, in the next part relation between the small world property and the quality of papers is analyzed.

**Table 5:** Regression results for average number of articles per author model.

Source	SS	df	MS		Number of obs	= 12
Model	.003751416	2	.001875708		F (2, 9)	= 9.84
Residual	.001715226	9	.000190581		Prob > F	= 0.0054
Total	.005466641	11	.000496967		R-squared	= 0.6862
					Adj R-squared	= 0.6165
					Root MSE	= .01381
**art_per_aut**	**Coef.**	**Std. Err.**	**t**	**P > |t|**	**[95% Conf. Interval]**	
*SW*	−.0000525	.0000133	−3.93	0.003	−.0000827	−.0000223
*netcon*	−.0005074	.000471	−1.08	0.309	−.0015730	.0005582
_*cons*	.4630127	.0224446	20.63	0.000	.4122395	.5137859

Two linear regression models were considered to check the relation between small world and quality of the publications, one is based on number of citations the articles received, and one on average impact factor of the journals in which the articles were published. Both mentioned measures can serve as a proxy for quality, but with a slightly different meaning. Impact factor indicates the respectability of the journal, *i*.*e*. the quality and the level of contribution perceived by the authors and the reviewers of the paper, whereas the citations show the impact of the article on the scientific community and on the subsequent research. Since both proxies have some flaws, it was decided to analyze both of them. Table 6 shows the regression results for the relation between the small world structure and the normalized number of citations received in the subsequent year. The number of citations was normalized based on the year of publication since generally older articles have higher total number of citations. According to the results, the linear regression is well fitted to our data. In addition, both variables are significant at the level of 95% confidence and based on the resulting R^2^ the independent variables are relatively good predictors of the dependent variable. Controlling for the network connectivity, small world property is positively related with the quality of the papers in the following year in terms of number of citations received. Hence, it can be said that researchers may benefit from the small world structure to exchange ideas more easily, and since they get connected to other researchers they can improve the quality of their work by internal referring among the team members and other researchers in the network. This is consistent with other studies that analyzed the impact of network centrality measures (not specifically small world properties) on the quality of the papers measured by number of citations and found positive relations (*e*.*g*. [43]).

**Table 6:** Linear regression results for number of citations model.

Source	SS	df	MS		Number of obs	= 12
Model	1.93930954	2	.969654768		F (2, 9)	= 14.98
Residual	.582490083	9	.06472112		Prob > F	= 0.0014
Total	2.52179962	11	.229254511		R-squared	= 0.7690
					Adj R-squared	= 0.7177
					Root MSE	= .2544
**ncit**	**Coef.**	**Std. Err.**	**t**	**P > |t|**	**[95% Conf. Interval]**	
*SW*	.0006591	.000246	2.68	0.025	.0001027	.0012155
*netcon*	.0348163	.0086805	4.01	0.003	.0151796	.0544529
_*cons*	.8278689	.4136143	2.00	0.076	−.1077916	1.763529

The same analysis was performed using a different proxy for the quality of the papers, namely the average impact factor of the journals in which the articles were published. According to Table 7, a significant positive relation is observed between the average journal impact factor and the small world structure. This along with our findings from Table 6 confirms the important role of the small world systems in supporting researchers to produce higher quality publications. From the results it can be said that although small world network may harm the average rate of publications, team members may benefit from such a system to increase the overall quality of their publications.

**Table 7:** Linear regression results for impact factor model.

Source	SS	df	MS		Number of obs	= 12
Model	19.6831928	2	9.8415964		F (2, 9)	= 14.27
Residual	6.20607683	9	.689564092		Prob > F	= 0.0016
Total	25.8892696	11	2.35356997		R-squared	= 0.7603
					Adj R-squared	= 0.7070
					Root MSE	= .8304
**avgjif**	**Coef.**	**Std. Err.**	**t**	**P > |t|**	**[95% Conf. Interval]**	
*SW*	.0037786	.0008029	4.71	0.001	.0019623	.0055948
*netcon*	.0383485	.0283341	1.35	0.209	−.0257476	.1024446
_*cons*	2.807872	1.350081	2.08	0.067	−.2462237	5.861967

## Conclusion

This study focused on the co-authorship network of the Canadian researchers in engineering and natural sciences and investigated the existence of the small world structure and its relation with the researchers’ productivity, quality of their publications, and their team size. Several previous studies analyzed different co-authorship networks and found correlations between network centrality measures and researchers’ productivity (*e*.*g*. [19,20,22,23]), however to our knowledge no study has focused specifically on the relation between the small world properties and quality of the publications and scientific team size.

Our results show that the examined network exhibits significant small world properties by having very high clustering coefficient in comparison with the random networks of the equal size while the path lengths are almost the same. The separation degree among scientists decreases to around five in the final period, when it becomes even lower than famous Milgram’s finding of six degrees of separation [62]. Hence, the networks in the final periods become more connected and the low path length among the researchers allows them to exchange knowledge more easily. Moreover, in comparison with most of the other co-authorship networks that have been studied, our examined co-authorship network has relatively larger clustering coefficient, smaller average path length, and larger proportion of the largest component. Specifically, the size of the largest component is critical since the path length (and consequently the small world measure) can be only calculated in the connected sub-network. Hence, this study benefited from the large share of its largest component to have better estimations of the small world variables. On the other hand, the enormous largest component in our examined co-authorship network may represent the fact that the core research activity is being done in an inter-connected large cluster of the researchers. Of course, the size of the largest component also depends on the nature of the research activity and the level of its interdisciplinarity.

The results show that although the small world structure has a positive relation with the total number of publications, it is negatively correlated with the average productivity of the researchers. Since a positive relation was observed between the small world and the researchers’ team size, it can be concluded that researchers may benefit from the small world properties to get familiar with other active researchers in their field and expand their scientific team. This team expansion can bring them several advantages such as internal referring, better and faster access to expertise and other resources, new sources of funding, *etc*. that will result in higher rate of publication for the whole team. But since the size of the team has grown up the average productivity will become lower. Therefore, it seems that even though a small world network could not be positively related to the individual productivity of the researchers, it might help them to invest their efforts in a more efficient way. Being involved in larger teams and getting in contact with other experts in the field allow them to not only gain new skills but also employ their skills more efficiently. Tighter collaboration among the team members can also create a synergy among them that will surely result in higher productivity of the team. The positive relation between the small world structure and the papers’ quality also supports the idea that the small world structure may facilitate more effective exchange of knowledge among the team members that may result in higher quality works. However, It is also possible that the reinforcement of the relation between scientific performance and small world structure be more dependent on the team size rather than on small world property. For example, van Raan [63] found a positive relation between team size and quality of research.

As discussed before, small world properties were reported to follow the form of an inverted U-shape [53]. According to our results, the Canadian natural science and engineering network has seen its latest pick in the period of [2006–2008], after which the article production started to decrease. Hence, according to Gulati *et al*. [53] a decreasing trend is predicted for the years after 2010, resembling an inverted U-shape curve. Considering the observed relations with the examined bibliometric measures it would be suggested to reassess the structure of the network periodically. In general, knowing the structure of the collaboration network and its relation with the performance measures may help the decision makers to set better strategies in supporting collaborative activities

## Limitations and Future Work

The main limitation was in regard with the sample size. The reason for the selection of the time interval of 1996 to 2010 was that SCOPUS has a weaker coverage before 1996. Moreover, articles need at least three years to be well cited and as a result the periods after 2010 were not included. Future work can address this limitation by using other databases with more number of observations. More observations would allow analyzing the interrelations between the small world property and other network centrality measures to assess the combined impacts.

Another limitation was in regard with the calculation of the small world variable for which the largest component was considered. Although there are some suggestions in the literature for overcoming this limitation (*e*.*g*. [9]), they could be applicable when the special purpose customizable software for social network analysis is available to code a program to calculate the small world indicator over the whole network. However, as mentioned before the proportion of the largest component in this study was larger than other similar studies, which allowed us to make more realistic estimates of the small world measures.

Furthermore, we were exposed to some limitations in measuring scientific collaboration among the researchers as we were unable to capture other links that might exist among the researchers like informal relationships. These types of connections are never recorded and thus cannot be quantified, but there are certainly some knowledge exchanges occurring in such associations that could affect the network performance. In addition, there are also some drawbacks in using ctableo-authorship as an indicator of collaboration since collaboration does not necessarily result in a joint article [64]. An example could be the case when two scientists cooperate together on a research project and then decide o publish their results separately [65]. Hence, future work can address this issue by taking other indicators into account. Finally, since the analysis presented in this document was performed at the aggregate level, a future research direction can use a large dataset to investigate the relation between network structure variables and researchers’ performance indicators at the individual level of researchers.
